# Serum leptin, C‐reactive protein, and cancer mortality in the NHANES III

**DOI:** 10.1002/cam4.570

**Published:** 2015-12-02

**Authors:** Wahyu Wulaningsih, Lars Holmberg, Tony Ng, Sabine Rohrmann, Mieke Van Hemelrijck

**Affiliations:** ^1^ Cancer Epidemiology GroupDivision of Cancer StudiesSchool of MedicineKing's College LondonLondonUnited Kingdom; ^2^ Department of Surgical SciencesUppsala UniversityUppsalaSweden; ^3^ Regional Cancer CentreUppsala UniversityUppsalaSweden; ^4^ Randall Division and Division of Cancer StudiesRichard Dimbleby Department of Cancer ResearchSchool of MedicineKing's College LondonLondonUnited Kingdom; ^5^ Division of Chronic Disease EpidemiologyEpidemiology, Biostatistics and Prevention InstituteUniversity of ZurichZurichSwitzerland

**Keywords:** Cancer, C‐reactive protein, leptin, mortality, prospective study

## Abstract

Adipokines, such as leptin, may affect cancer through its link with inflammation and obesity. We investigated the association between leptin, C‐reactive protein, and risk of cancer death while accounting general and abdominal obesity. From the Third National Health and Examination Survey (NHANES III), we selected 5957 adult men and women with baseline measurements of serum leptin and CRP. Multivariable Cox regression was used to assess leptin and CRP levels (low, moderate, high) in relation to risk of cancer death. Stratification analyses were performed for obesity as defined by body mass index (BMI) and waist circumference. Fine and Gray regression was performed to account for death from cardiovascular disease and other causes as competing events. A total of 385 participants died of cancer during a mean follow‐up of 18 years. After adjusting for BMI and waist circumference, an inverse association with log‐transformed leptin was found for women, with a hazard ratio (HR) of 0.81 (95% confidence interval [CI]: 0.51–1.30) and 0.40 (95% CI: 0.24–0.68) for moderate and high compared to low levels of leptin, respectively; *P*_trend_ = 0.0007). No association for leptin was observed in men, but higher CRP corresponded to increased risk of dying from cancer (HR: 2.98; 95% CI: 1.57–5.64 for the highest vs. lowest categories of CRP). Similar associations were observed with competing risk analysis also adjusted for BMI and waist circumference. Contrasting associations of serum leptin and CRP with cancer mortality may indicate sex‐specific biological or environmental pathways linking obesity and cancer in men and women which warrant mechanistic investigations.

## Introduction

Leptin is one of the most important hormones secreted by the adipocytes 1. Besides regulating food intake and energy expenditure 2, leptin plays an essential role in hematopoiesis, reproductive function, and glucose and lipid metabolism 3. More recently, leptin has also been linked to cancer 4. Enhanced expressions of leptin and its receptor (Ob‐R) are found in solid cancers including breast and ovarian cancers, and have been associated to metastasis and poor prognosis 5, 6. However, conflicting evidence exists in the context of cancer incidence. For instance, in a meta analysis comprising 23 case–control studies, a protective effect of serum leptin against postmenopausal breast cancer was reported 7. In contrast, a positive association was seen in recent nested case–control studies, where serum leptin was measured prospectively prior to diagnosis in breast cancer cases 8, 9. Meanwhile, circulating Ob‐R has been linked to a lower risk of colorectal cancer in a nested case–control study despite a null finding for leptin 10. These inconsistent findings may reflect an involvement of other factors in the relationship between leptin and carcinogenesis, as well as potential time‐sensitivity of this association.

Obesity may promote the development of cancer 11, but their mechanistic association remains unclear. There is indication that chronic inflammation may mediate obesity and cancer 12. Interestingly, a role of leptin in inflammation has been suggested 13, as shown by a linear association between leptin and markers of inflammation 14. Both increased inflammatory activity and leptin production are common features of obesity 15, thus it remains unclear whether pathways linking obesity and the development of cancer involve leptin production or inflammation, or whether there are simultaneous effects of these two processes on cancer susceptibility.

Presently, there is lack of observational studies assessing leptin in relation to cancer while accounting for inflammation and different definitions of obesity. Therefore, we sought to disentangle this complex association between leptin, inflammation and cancer by assessing serum levels of leptin and C‐reactive protein (CRP), a common inflammatory marker 16, in relation to cancer mortality in the Third National Health and Nutrition Examination Survey (NHANES III) while accounting for general and abdominal obesity. Additionally, since both markers are linked to death from cardiovascular disease 17, we used cardiovascular mortality as a competing outcome in our analysis.

## Methods

### Study population

The National Center for Health Statistics (NCHS) conducted NHANES III between 1988 and 1994 and designed it as a multistage stratified, clustered probability sample of the U.S. civilian noninstitutionalized population who was at least 2 months old. All subjects participated in an interview conducted at home and an extensive physical examination, which included a blood sample taken in a mobile examination center 18. Despite a cross‐sectional design, mortality follow‐up was provided by the NCHS through December 31, 2011, allowing the use of the dataset as a prospective cohort 19. From recruited NHANES III participants, we selected 5957 men and women aged 20 and over who had baseline measurements of serum leptin and CRP, available information on body mass index (BMI) and waist circumference, and for whom follow‐up information was available. No participant reported a history of any cancer at the baseline interview. The protocols for the conduct of NHANES III were approved by the Institutional Review Board of the NCHS, Centers for Disease Control and Prevention. Written informed consent was obtained from all participants 18.

### Serum leptin and CRP measurements

Serum specimens were stored at −70°C and went through at least one freeze–thaw cycle during a mean of 8 year of storage before leptin concentrations were measured. Serum leptin was measured by radioimmunoassay at Linco Research, Inc. (St Charles, MO) 20. The minimum detectable concentration of the assay is 0.5 ng/mL. Within‐ and between‐assays coefficients of variation were <5%. Levels of serum leptin were categorized into low, moderate, and high based on sex‐specific tertiles 21, with cut‐off points of 3.3 and 6.3 *μ*g/L for men and 10.8 and 20 *μ*g/L for women. Serum CRP was measured with an automated Behring Nephelometer Analyzer System (Behring Diagnostics, Inc, Somerville, NJ) 22. Coefficients of variation ranged from 3.2 to 16.0% throughout data collection. Tests were repeated for specimens with results of >10 mg/L. Because levels of CRP below 2.2 mg/L were undetectable in the NHANES III, we used clinical cut‐off points as previously described 16: low (<2.2 mg/L), moderate (2.2–10 mg/L), and high (≥10 mg/L).

### Covariates

Information on age (years), race/ethnicity (non‐Hispanic white, non‐Hispanic black, Mexican American, and other), cigarette smoking (never, former, and current smokers), alcohol consumption (never, up to once/week, 2–3 times/week, 4–6 times/week, daily or more), vigorous physical activity (yes, no), and self‐reported history of cancer (yes, no) was collected during the interview. Socioeconomic status was estimated with poverty‐to‐income ratio (PIR), a ratio of total family income to the official poverty threshold at the family level. A PIR <1 indicated that income was less than the level of poverty. We categorized PIR in this study into <1, 1–2, and ≥2.

### Obesity status

Body measurements were performed using standardized methods and equipment 23. Weight was measured in pounds and automatically converted to kilograms with an electronic weight scale. Participants only wore underwear, disposable paper gowns, and foam rubber slippers. Standing height was measured with a fixed stadiometer to the nearest 1 mm. Body mass index (BMI) was calculated as weight in kilograms divided by the square of the height in meters. Waist circumference was measured at the high point of the iliac crest at minimal respiration using a steel measuring tape to the nearest 1 mm 23. General obesity (obese, not obese) was defined as having a BMI of 30 kg/m^2^ or more 24. Abdominal obesity (obese, not obese) was defined as waist circumference of >102 cm in men and >88 cm in women 25.

### Mortality and follow‐up

Information on dates and causes of death was obtained from data linkage of the NHANES dataset with the National Death Index (NDI). This linkage was performed by the NCHS through probabilistic matching with social security number, birth date, occupation, and other personal data, and confirmation with death certificate when possible 19. Follow‐up time was calculated from interview date/examination date until date of death or end of study (31 December, 2011), whichever came first. Underlying causes of death were based on ICD‐9 codes through 1998 and on International Classification of Diseases, 10th version (ICD‐10) codes for deaths occurring after 1998. In order to adjust for changes between the two coding systems, final cause of deaths occurring prior to 1999 were re‐coded into comparable ICD‐10‐based underlying cause of death groups 19. The primary outcome of this study was cancer‐specific death (ICD‐10: C00‐C97). Only aggregate information on leading causes of death is available in the 2011 mortality follow‐up, thus rendering analysis by specific cancer sites not possible. Death from major cardiovascular diseases (ICD‐10: I00‐I09, I11, I13, I20‐I51, I60‐I69) and other causes were assessed as competing outcomes.

### Statistical analysis

Sampling weights for NHANES III were used to account for sampling variability and to adjust for differential probability of selection of persons 18. Due to differential distribution of serum leptin in men and women, we performed our analysis for men and women separately. Cox proportional hazards regression was used to assess risk of cancer death by categories of CRP and leptin. A test for trend was conducted by using assignment to categories as an ordinal scale. First, we carried out our analyses using two multivariable models: the first was adjusted for age, race/ethnicity, PIR, tobacco smoking, alcohol consumption, and vigorous physical activity. The final model included BMI and waist circumference to account for the effects of obesity. A test for multiplicative interaction between leptin and CRP was performed based on the suggested correlation between the two variables 21. To further elucidate potential effect modification by obesity 16, we stratified our analysis based on general obesity status while adjusting for waist circumference, and by abdominal obesity while adjusting for BMI. In addition to interaction between leptin and CRP, we also assessed the interaction of each marker with obesity status. Finally, since the association of both markers and cardiovascular death 17 may affect their impact on cancer mortality, we performed Fine and Gray regression with deaths from major cardiovascular diseases and other causes as competing outcomes. The Fine and Gray analysis has been used to predict cumulative incidence of primary outcome in presence of competing outcomes, which may have created a competing risks situation 26. We treated categories of leptin and CRP as ordinal variables and adjusted the models for age, race/ethnicity, PIR, tobacco smoking, alcohol consumption, vigorous physical activity, BMI, and waist circumference. Statistical significance was defined as two‐sided *P*‐values <0.05. All analyses were conducted with SAS release 9.3 (SAS Institute, Cary, NC) and R version 3.1.0 (R Foundation for Statistical Computing, Vienna, Austria).

## Results

**Figure 1:**
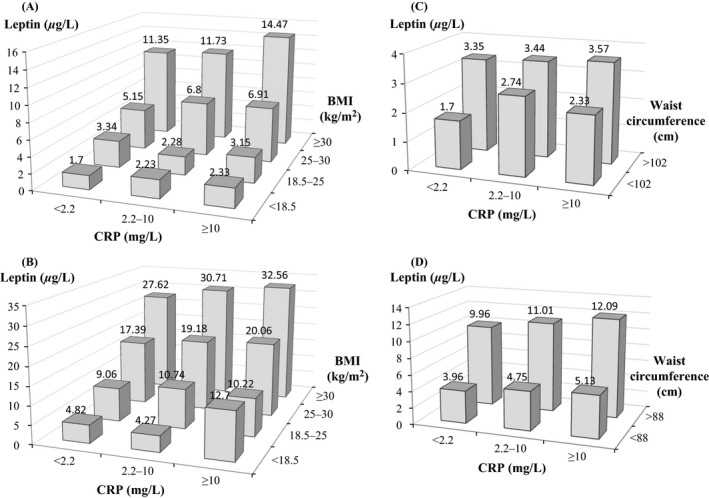
Serum concentrations of leptin by C‐reactive protein (CRP) clinical cut‐offs and body mass index in men (A) and women (B), and by CRP clinical cut‐offs and waist circumference in men (C) and women (D).

During a mean follow‐up of 18 years, a total of 385 participants died of cancer and 507 from major cardiovascular diseases. Table 1 showed weighted characteristics of study participants by sex. Overall, increased leptin levels were observed in men and women following higher categories of CRP, BMI, and waist circumference (Fig. 1), with the highest concentrations of leptin seen in men and women with high CRP and BMI ≥ 30 kg/m^2^ or the upper category of waist circumference.

**Table 1:** Weighted characteristics of study population by sex

	Men (*N *=* *2759)	Women (*N *=* *3198)
Age (years) – Mean (SD)	42.64 (0.55)	43.81 (0.60)
Follow‐up (years) – Median (IQR^a^)	19.35 (17.53–20.95)	19.38 (17.65–20.94)
Race – Ethnicity (%)		
Non‐Hispanic white	76.52	76.42
Non‐Hispanic black	9.77	11.32
Mexican American	5.56	4.78
Other	8.16	7.48
Poverty‐to‐income ratio		
<1	16.34	19.89
1–2	19.09	19.97
≥2	64.57	60.14
Alcohol consumption (%)		
Never	35.09	51.69
Up to once/week	18.15	21.28
2–3 times/week	16.50	13.11
4–6 times/week	15.44	8.19
Daily or more	14.82	5.73
Smoking behavior (%)		
Never	36.36	55.53
Former	32.29	20.13
Current	31.35	24.34
Vigorous Physical activity (%)	11.47	8.48
Waist circumference (cm) – Mean (SD)	95.50 (0.48)	88.22 (0.47)
Body mass index (kg/m^2^)		
<18.5	0.68	2.981
18.5–25	38.57	46.04
25–30	40.46	27.06
≥30	20.28	24.09
Cancer death (%)	4.72	5.15
Cardiovascular death (%)	6.34	4.80

a Interquartile range.

When we examined the association between serum leptin or CRP and cancer death with the first model, high levels of CRP in men corresponded to higher risk of dying from cancer, with a hazard ratio (HR) of 2.98 and 95% confidence interval (CI) of 1.57 to 5.64 for the highest category of CRP compared to the lowest. No association was observed between leptin and cancer death in both sexes (Table 2). We further adjusted this model for obesity indicators, BMI and waist circumference, and no changes were seen with CRP. However, this revealed a marked inverse association between serum leptin and risk of cancer death in women (HR: 0.81; 95% CI: 0.51–1.30 and 0.40; 95% CI: 0.51–1.30 for moderate and high compared to low levels of leptin, respectively; *P*_trend_ = 0.0007). No interaction between categories of leptin and CRP was observed (Table 2).

**Table 2:** Sex‐specific associations of serum leptin and C‐reactive protein (CRP) with cancer death in the NHANES III

	*N* cancer death/*N* total	HR (95% CI)	
		Model 1^a^	Model 2^b^
*Men*			
Leptin (*μ*g/L)			
Low	49/909	1.0 (Reference)	1.0 (Reference)
Moderate	71/962	0.87 (0.51–1.49)	0.75 (0.43–1.35)
High	79/964	0.86 (0.50–1.47)	0.60 (0.26–1.33)
*P*_trend_		0.60	0.22
CRP (mg/L)			
Low	195/2047	1.0 (Reference)	1.0 (Reference)
Moderate	56/596	1.33 (0.82–2.18)	1.31 (0.85–2.04)
High	34/192	2.98 (1.57–5.64)	2.90 (1.52–5.53)
*P*_trend_		0.003	0.002
*P*_interaction_ leptin and CRP		0.13	0.13
*Women*			
Leptin (*μ*g/L)			
Low	54/1081	1.0 (Reference)	1.0 (Reference)
Moderate	71/1096	1.19 (0.74–1.91)	0.81 (0.51–1.30)
High	61/1128	0.93 (0.57–1.51)	0.40 (0.24–0.68)
*P*_trend_		0.80	0.0007
CRP (mg/L)			
Low	103/2027	1.0 (Reference)	1.0 (Reference)
Moderate	58/914	1.41 (0.84–2.38)	1.19 (0.67–2.11)
High	25/364	1.10 (0.61–1.99)	0.86 (0.45–1.63)
*P*_trend_		0.28	0.99
*P*_interaction_ leptin and CRP		0.72	0.81

a Adjusted for age (continuous) and waist circumference, race/ethnicity, poverty‐to‐income ratio (PIR), tobacco smoking, alcohol consumption, and vigorous physical activity.

b Adjusted for age (continuous) and waist circumference, race/ethnicity, PIR, tobacco smoking, alcohol consumption, vigorous physical activity, body mass index (continuous) and waist circumference (continuous), and waist circumference.

We sought to further unpick the effect of obesity by stratification analyses based on general and abdominal obesity status. In men, no association between serum leptin and cancer death was observed (Table 3). For CRP, higher levels in men without general obesity was observed (HR: 3.62; 95% CI: 1.86–7.04 for high compared to low CRP, *P*_trend_ = 0.004). Interestingly, a higher risk of cancer death with the highest CRP levels was also seen in men with abdominal obesity (HR: 3.43; 95% CI: 1.03–11.48 compared to low CRP) but not those without. Nevertheless, no significant interaction between each definition of obesity and CRP was found. On the other hand, higher serum leptin was inversely associated with cancer death in women with or without general obesity, for example, HR among women with BMI <30 kg/m^2^ was 0.76 (95% 0.44–1.32) and 0.30 (0.14–0.65) for moderate and high levels compared to low leptin, respectively (*P*_trend_ = 0.003). Results were less clear in stratification by waist circumference, or when CRP was assessed (Table 3). No strong interaction between leptin and CRP was suggested, although among men with abdominal obesity, interaction approached statistical significance (*P* = 0.07). Nevertheless, in a follow‐up analysis when we included leptin and CRP in the same model, similar findings were observed.

**Table 3:** Sex‐specific associations of serum leptin and C‐reactive protein (CRP) with cancer death in the NHANES III, stratified by obesity status. All models were adjusted for age, race/ethnicity, poverty‐to‐income ratio (PIR), tobacco smoking, alcohol consumption, and vigorous physical activity

	General obesity^a^		Abdominal obesity^b^	
	Not obese	Obese	Not obese	Obese
*Men*				
*N* cancer death/*N* total	145/2177	43/582	114/1965	74/794
Leptin (*μ*g/L)				
Low	1.0 (Reference)	1.0 (Reference)	1.0 (Reference)	1.0 (Reference)
Moderate	0.85 (0.50–1.44)	1.18 (0.22–6.40)	0.91 (0.47–1.76)	4.09 (0.88–19.10)
High	0.61 (0.24–1.53)	1.56 (0.35–6.94)	0.64 (0.20–1.99)	2.71 (0.64–11.45)
* P*_trend_	0.28	0.56	0.49	0.59
*P*_interaction_ leptin and obesity		0.52		0.43
CRP (mg/L)				
Low	1.0 (Reference)	1.0 (Reference)	1.0 (Reference)	1.0 (Reference)
Moderate	0.99 (0.52–1.89)	1.48 (0.41–5.40)	1.06 (0.57–1.97)	1.33 (0.63–2.79)
High	3.62 (1.86–7.04)	0.59 (0.13–2.72)	2.22 (0.97–5.05)	3.43 (1.03–11.48)
*P*_trend_	0.004	0.89	0.17	0.07
*P*_interaction_ CRP and obesity		0.29		0.64
*P*_interaction_ leptin and CRP	0.39	0.09	0.11	0.08
*Women*				
*N* cancer death/*N* total	112/2235	67/963	52/1438	127/1760
Leptin (*μ*g/L)				
Low	1.0 (Reference)	1.0 (Reference)	1.0 (Reference)	1.0 (Reference)
Moderate	0.76 (0.44–1.32)	1.77 (0.20–16.16)	1.06 (0.49–2.30)	1.24 (0.64–2.41)
High	0.30 (0.14–0.65)	0.65 (0.08–5.08)	N/A	1.07 (0.55–2.07)
* P*_trend_	0.003	0.01	0.15	0.09
*P*_interaction_ leptin and obesity		0.63		0.41
CRP (mg/L)				
Low	1.0 (Reference)	1.0 (Reference)	1.0 (Reference)	1.0 (Reference)
Moderate	1.32 (0.66–2.63)	0.89 (0.33–2.36)	1.00 (0.47–2.11)	1.29 (0.70–2.36)
High	0.65 (0.25–1.68)	0.92 (0.43–1.94)	N/A	1.14 (0.62–2.07)
* P*_trend_	0.78	0.89	0.40	0.63
*P*_interaction_ CRP and obesity		0.83		0.35
*P*_interaction_ leptin and CRP	0.77	0.37	0.92	0.46

a Adjusted for waist circumference (continuous).

b Adjusted for body mass index (continuous).

Finally, to account for competing risks, we ran Fine and Gray regression to estimate cumulative mortality of cancer over time with levels of leptin or CRP as the predictor variable and deaths from major cardiovascular diseases and other causes as competing outcomes. The analysis was adjusted for all potential confounders including BMI and waist circumference. Men with higher CRP were shown to have higher cumulative mortality from cancer over time (*P* = 0.009), whereas no association for serum leptin was found (*P*_trend_ = 0.17). In women, serum CRP was not suggested to correlate with cancer mortality (*P* = 0.59). On the other hand, higher cumulative mortality from cancer was noted in women with higher serum leptin (*P* = 0.006). Therefore, our results from the competing risk analysis corroborated our findings from Cox regression models.

## Discussion

This study was based on a nationally representative sample of the U.S. population. We observed a protective effect of leptin against cancer death in women and higher cancer death with increased CRP in men. No marked interaction between leptin and CRP was found. Similar associations were observed when competing risk analyses with deaths from major cardiovascular diseases and other causes as competing outcomes were employed.

Proposed mechanisms linking leptin and carcinogenesis mostly suggest that higher leptin exposure increases predisposition to the disease 3. The long isoform of leptin receptor (Ob‐R) is similar to a type I cytokine receptor, with an ability to activate downstream JAK/STAT signaling pathway, a known transcription activator for genes involved in cell proliferation, survival, angiogenesis, and metastasis 27. Furthermore, the activation of ObR may lead to phosphorylation of insulin receptor substrate (IRS‐1), initiating activation of PI3K/Akt pathway, which is also important in carcinogenesis 28. Besides directly eliciting cancer‐related signaling, leptin also displays proinflammatory properties 29. Inflammation may also promote cancer by activation of signaling molecules including STAT3 and NF‐*κ*B 30. Despite suggestive experimental findings, there is limited observational evidence documenting the importance of leptin‐inflammation interplay in cancer incidence or mortality.

Findings from population‐based studies for the link between leptin and cancer are scarce. Some evidence suggests a positive association between prediagnostic serum leptin and risk of cancer for colorectal 31, breast 8, prostate 32, and endometrial cancer 33, as well as renal cell carcinoma 4, but results are contradictory 34, 35. In a large nested case–control study based on the European Prospective Investigation into Cancer and Nutrition (EPIC), no association was reported between serum leptin and risk of colorectal cancer regardless further adjustment for BMI (RR: 0.85 (95% CI: 0.56–1.29) for the highest quintile compared to the lowest; *P*_trend_ = 0.76) 10. We found a lack of association between leptin and cancer death, whereas CRP was positively associated to cancer death in men. The inverse association for leptin, which was not observed in previous studies such as the EPIC Study focusing on colorectal cancer 10 might be attributed to the use of cancer mortality as an outcome instead of cancer incidence. Therefore, it is possible that leptin, despite being weakly associated to cancer incidence, may reflect susceptibility for fatal malignancies.

With respect to obesity, leptin and inflammation have gained increasing interest with regards to their potential implications in cancer development 12. Leptin resistance may occur in obesity 36, where higher levels of leptin follow. Interestingly, CRP has been identified as one of the major serum leptin‐interacting proteins (SLIPs) which may worsen leptin resistance 37 and support a biological interaction between leptin and CRP in the context of diseases. In this study, a nearly statistically significant interaction between leptin and CRP was observed in men with abdominal obesity. Romero‐Corral and colleagues 21 stated that such interaction occurs when assessing cardiovascular disease, resulting in a weaker association between CRP and cardiovascular disease after adjustment for leptin. Nevertheless, we did not observe any alteration in our findings after leptin and CRP were both included in the same analysis, thus suggesting minimal interaction between serum leptin and CRP with respect to cancer mortality as an outcome.

In many studies including ours, serum concentrations of leptin are positively correlated with that of CRP regardless of obesity 38, 39. However, we observed different effects between leptin and CRP levels on cancer mortality. This may signify differential roles between leptin and CRP in the scope of cancer, which require further biological investigations. From the competing risk analysis where we took into account competing outcomes, it was further suggested the inverse association between serum leptin and cumulative mortality from cancer in women, and a positive association for CRP in men. Our findings therefore indicate that the associations between leptin or CRP and cancer may be unique and do not resemble the additive effects observed with cardiovascular disease 21. This interesting observation may suggest different biological pathways linking leptin and inflammation with cancer, which may involve sex‐specific biological and environmental factors. Such observations thus call for further investigations to assess specific cancers and relevant mechanistic approaches.

This strength of this study is its generalizability following the use of nationally representative data of the U.S. population. We were able to adjust for potential confounders and stratify by overweight status. To our knowledge, this is the first study investigating the interaction between leptin and CRP in relation to cancer in the population. A limitation of this study is that there was no information on cancer incidence, so that we were only able to assess these markers in relation to cancer mortality. In the NHANES, information on causes of death was collected by means of probabilistic matching 19. Although we only selected those considered to have eligible mortality status, potential misclassification may have occurred. Low number of cases also hampered our stratification analyses, and therefore future studies with sufficient number of cases are necessary to further investigate this topic. Additionally, our analyses relied on a single measurement so that it may be prone to measurement error and within‐person variation. The laboratory methods used for CRP measurement at the time the NHANES III was conducted were unable to perform a high sensitivity assay of this marker. Nevertheless, serum CRP in the NHANES III population was reported to be associated to CRP‐related genetic variation 40, justifying the usefulness of this marker despite its limitation in quantitatively measuring low levels of CRP. Finally, abnormal levels of leptin and CRP may occur secondary to cancer which may result in reverse causation. We have excluded participants with cancer at baseline, however, residual confounding may have occurred.

## Conclusion

Our study showed that leptin may be inversely associated with cancer mortality in women, and CRP corresponded with higher risk for cancer death in men. Interaction between CRP and leptin is likely to be minimal in the study on cancer mortality, unlike previous evidence suggested in cardiovascular disease. It is imperial for further studies to address the discrepancies in effects on cancer between adipokines and inflammatory markers in order to fully comprehend the mechanism linking obesity‐related features and carcinogenesis. Furthermore, the differential associations with cancer death between men and women may point toward their potential use in future risk modification strategies targeting mortality from cancer.

## Conflict of Interest

No conflict of interest declared.
